# Discrepancy between reference phenotypic and genotypic detection of *cphA* metallo-β-lactamase in *Aeromonas* spp.

**DOI:** 10.1128/jcm.01272-25

**Published:** 2026-04-30

**Authors:** Benjamin von Bredow, Marisol Trejo, Kevin W. Ward, Kevin P. Trieu, Cynthia A. Santellan, Omai B. Garner, Shangxin Yang, Sukantha Chandrasekaran

**Affiliations:** 1 Department of Pathology and Laboratory Medicine, Oakland University William Beaumont School of Medicine 159878, Auburn Hills, Michigan, USA; 2 Department of Pathology and Laboratory Medicine, UCLA David Geffen School of Medicine, University of California, Los Angeles 166650 , Los Angeles, California, USA; 3 Department of Pathology and Laboratory Medicine, University of California, Los Angeles 166650 , Los Angeles, California, USA; 4 Ventura County Public Health Laboratory 8190 , Oxnard, California, USA; 5 Department of Medicine, UCLA David Geffen School of Medicine, University of California 664527, Los Angeles, California, USA; Cleveland Clinic, Cleveland, Ohio, USA

**Keywords:** mCIM, carbapenemase, antibiotic susceptibility testing, *Aeromonas*

## Abstract

Infection with *Aeromonas* spp. can be difficult to treat due to the common presence of multiple β-lactamase genes. Although the presence of β-lactamase genes appears to be mostly species-specific, species-level identification of *Aeromonas* spp. is currently unreliable. Routine phenotypic susceptibility testing is unreliable for the detection of chromosomal *cphA* metallo-β-lactamase-mediated carbapenem resistance, which can lead to adverse outcomes. A reliable, easy-to-use phenotypic method for the detection of carbapenem resistance in *Aeromonas* spp. would allow laboratories to better guide treatment of these organisms. This study used whole-genome sequencing for the detection of β-lactamase genes in 43 *Aeromonas* spp. isolates as a reference to evaluate different phenotypic methods for the detection of *cphA*. Standard Kirby Bauer disk diffusion (KB) and broth microdilution (BMD) were least sensitive, detecting carbapenem resistance in 3 to 5 of 24 *cphA*-positive isolates, depending on the drug and method. Increased-inoculum KB detected resistance in 3 to 14 of 24 isolates, whereas increased-inoculum BMD detected resistance in 11 to 18 of 24 isolates depending on the carbapenem. The modified carbapenem inactivation method (mCIM) was positive for all 24 isolates, whereas EDTA-modified mCIM was positive for 19/24 isolates. None of the 19 *cphA*-negative isolates tested positive by mCIM or resistant by any phenotypic method. The mCIM is an easy-to-implement method that detected *cphA*-mediated carbapenem resistance with 100% sensitivity and specificity and could help guide effective treatment of *Aeromonas* sp. infection.

**IMPORTANCE:**

Serious infections with *Aeromonas* species are associated with high rates of morbidity and mortality. Some species can have a carbapenemase that is not detectable by typical phenotypic methods. It is important for clinical laboratories to be aware and have a method for identifying and reporting this phenotype. Clinicians should also be aware of this resistance phenotype when choosing an antibiotic for therapy. Here, we show the difficulty in using broth microdilution or disk diffusion to identify carbapenem resistance mediated by a specific carbapenemase, *cphA*, in *Aeromonas* spp. Through genotypic analysis, we also found *cphA* to be associated with certain species of *Aeromonas*. Unfortunately, identification to the species level using typical methods like MALDI-TOF is also difficult. This study presents data for a previously well-established method, modified carbapenem inactivation method, that can be utilized to reliably detect this resistance phenotype in commonly encountered *Aeromonas* species.

## INTRODUCTION

*Aeromonas* spp. are Gram-negative facultative anaerobic bacteria that are typically found in freshwater environments, including lakes, rivers, and groundwater, but can also survive in brackish seawater and soil and can colonize animals and contaminate food products (1, 2). *Aeromonas* spp. cause gastroenteritis and are associated with infections following medicinal leech therapy (3, 4). Wound infections with *Aeromonas* spp. can develop after traumatic injury with freshwater exposure and can range from uncomplicated cellulitis to severe or life-threatening presentations, including myonecrosis and necrotizing fasciitis (5). Systemic infections caused by *Aeromonas* spp. include hepatobiliary infections, peritonitis, and septicemia (3, 6).

Whereas gastroenteritis caused by *Aeromonas* spp. is generally self-limiting, treatment of extraintestinal *Aeromonas* sp. infection can be challenging. *Aeromonas* spp. express a variety of chromosomal β-lactamases, including Ambler Class B, Class C, and Class D β-lactamases (7). Antimicrobial susceptibility testing is required to guide appropriate therapy, as different combinations of β-lactamases result in different susceptibility patterns, and resistance to fluoroquinolones in *Aeromonas* spp. is increasing (8). One of the most concerning β-lactamases is the Class B metallo-β-lactamase, *cphA*. This gene is commonly found in *Aeromonas* species (9–11) and has a very specific substrate profile targeting carbapenems only (12–14). The presence of the different classes of β-lactamases has been shown to be species-specific (9), with almost all *A. hydrophila, A. veronii,* and *A. dhakensis* carrying this gene, whereas it is generally absent from *A. caviae* (12–14). However, accurate species identification of *Aeromonas* spp. can be challenging. MALDI-TOF and sequencing of the *rrs* (16S rRNA) gene alone have not been able to reliably identify *Aeromonas* spp. to species level (11, 15). These issues together make susceptibility predictions challenging.

Carbapenems may be used to treat serious or polymicrobial infections with *Aeromonas* spp., but phenotypic susceptibility testing has shown limited sensitivity for the detection of carbapenem resistance mediated by a *cphA*-class metallo-β-lactamase (MBL). Failure to detect *cphA*-mediated carbapenem resistance by phenotypic methods has been reported to result in adverse outcomes (11, 16). According to CLSI and EUCAST, antimicrobial susceptibility testing of *Aeromonas* spp. may be performed by broth microdilution (BMD) and Kirby-Bauer disk diffusion (KB), but previous studies have shown that these methods may fail to detect *cphA*-mediated carbapenem resistance (11, 16). Attempts to increase the accuracy of phenotypic methods by increasing the inoculum size have had some success (9, 13). The Clinical and Laboratory Standards Institute (CLSI) currently recommends the modified carbapenem inactivation method (mCIM) for phenotypic detection of carbapenemases in the *Enterobacterales* and *Pseudomonas aeruginosa* (17), and following a positive mCIM, an EDTA-modified mCIM (eCIM) can be performed to differentiate MBL from other classes of carbapenemase in the Enterobacterales (17). Although these tests are only endorsed by CLSI for use in the order of *Enterobacterales* (and *P. aeruginosa* for mCIM only), researchers have evaluated their use in other organisms, with varying results (18–22).

In this study, we use whole-genome sequencing (WGS) to accurately identify *Aeromonas* spp. to the species level and genotypically determine the presence of different classes of β-lactamases, including *cphA*. We further evaluated the ability of different phenotypic methods to predict the presence of *cphA*, including Kirby-Bauer Disk Diffusion (KB), broth microdilution (BMD), and the modified carbapenem inactivation method without (mCIM) and with EDTA (eCIM).

## MATERIALS AND METHODS

Isolates of *Aeromonas* spp. recovered and identified during routine bacterial culture of patient specimens submitted to our clinical laboratory between 2018 and 2024 were included in this study. Only one isolate was included from each patient. Routine species identification was performed using Vitek MS MALDI-TOF technology using the FDA cleared library (Database Version 3.2). Whole-genome sequencing of isolates was performed as previously described (23). In brief, bacterial DNA was extracted using the EZ1 DNA Tissue Kit (QIAGEN), sequencing libraries were prepared using the Illumina DNA Prep kit (Illumina), followed by sequencing using the MiSeq Reagent Kit v2 (Illumina).

Sequence data were analyzed using the CLC Genomics Workbench (QIAGEN), as previously described (23). Consensus *rrs, rpoB,* and *groEL* gene sequences were generated and used to identify the isolates to the species level using a published and clinically validated test (23). In brief, greater than or equal to 99% identity to the three genes must be achieved. For species that could not be identified by the standard algorithm, the *recA* gene sequence was used for analysis, as previously described (24). Species identification was further confirmed by phylogenetic analysis, evaluating clustering of study isolates with reference sequences using a K-mer tree (Fig. S1). The method used for analysis is feature frequency profile, which is an alignment-free genome comparison using a Kmer length of 16 with the prefix ATGAC using both strands (25). Resistance genes were identified using the QIAGEN Microbial Insights AR (QMI-AR) database. The CLC QMI-AR database combines four publicly available databases, including ARGAnnot (26), NCBI ResFinder (27), and CARD (28). Resistance Database QMI-AR Nucleotide Database Version and 2021-08 and 7.0 (2025) was utilized.

Phenotypic antimicrobial resistance (AMR) testing was performed and interpreted according to CLSI recommendations for *Aeromonas* spp (29). Standard and increased bacterial inoculum was used, as prior studies have shown that increasing the bacterial inoculum may increase detection of cphA-mediated carbapenem resistance (9, 13). Reference broth microdilution (BMD) was performed using both standard 0.5 McFarland (McF) and 3 McF inocula. Kirby-Bauer Disk Diffusion (KB) was performed using standard 0.5 McF and 1.5 McF inocula. Interpretive criteria from CLSI’s M45 third edition are shown in Table 1. Standard CLSI breakpoints, which are established using a 0.5 McF, were applied to both the 0.5 and increased (1.5 and 3 McF) inocula. The modified carbapenem inactivation method (mCIM) and mCIM with EDTA (eCIM) were performed and interpreted according to standard methods for the detection of carbapenemases and MBLs in the *Enterobacterales*, respectively (17).

**TABLE 1:** Interpretive criteria applied^*a*^

	Kirby-Bauer zone size (mm)			Microbroth dilution (µg/mL)		
Drug	S	I	R	S	I	R
Ertapenem	≥22	19–21	≤18	≤0.5	1	≥2
Meropenem	≥23	20–22	≤19	≤1	2	≥4
Imipenem	≥23	20–22	≤19	≤1	2	≥4

^*a*^ CLSI. Methods for Antimicrobial Dilution and Disk Susceptibility testing of Infrequently Isolates or Fastidious Bacteria. 3rd. ed. CLSI Guideline M45. Wayne, PA; Clinical Laboratory Standards Institute; 2015.

## RESULTS

Forty-three isolates of *Aeromonas* spp. were included in the study. These isolates were derived from a variety of specimen types, including blood (19 specimens), urine (4), wound (6), stool (4), respiratory (5), and body fluids (5). Detailed specimen descriptions are listed in Table S1. The entire genome of each isolate was sequenced to identify to the species level as well as identify antibiotic resistance genes (Table S1). Specimen source, genotypic identification, and detected β-lactamase genes for each isolate are shown in Table 2. Eighteen isolates were identified as *A. caviae*, 13 were identified as *A. veronii*, 1 was identified as *A. media,* and 9 as *A. hydrophila*. Two isolates were identified as either *A. dhakensis* or *A. hydrophila*. The taxonomy of *A. dhakensis* is complex, and the species has been described as synonymous with *A. hydrophila* subsp. *dhakensis* and *A. aquariorum*. Additional analysis of the *recA* gene showed the closest match for this isolate to be *A. hydrophila* subsp. *dhakensis*, followed by a list of sequences representing *A. hydrophila, A. dhakensis,* and *A. aquariorum*. It is therefore likely that the isolate is *A. dhakensis,* and discrepant identifications represent the taxonomic complexity of the species. Species level identification was further confirmed using phylogenetic clustering with reference genomes. These results are presented in Fig. S1. All isolates cluster with the reference genome matching their identification by WGS.

**TABLE 2:** Specimen source, species, and beta-lactamases encoded by study isolates

ID	Source	Species	CLASS A β-lactamase	CLASS B β-lactamase	CLASS C β-lactamase	CLASS D β-lactamase
1	Blood	*A. caviae*			blaMOX	blaOXA
2	Blood	*A. caviae*			blaMOX	blaOXA
3	Blood	*A. caviae*			blaMOX	blaOXA
4	Blood	*A. caviae*			blaMOX	blaOXA
5	Urine	*A. caviae*			blaMOX	blaOXA
6	Urine	*A. caviae*			blaMOX	blaOXA
7	Respiratory	*A. caviae*			blaMOX	blaOXA
8	Stool	*A. caviae*			blaMOX	blaOXA
9	Wound	*A. caviae*			blaMOX	blaOXA (two types)
10	Blood	*A. caviae*			blaMOX	blaOXA
11	Respiratory	*A. caviae*			blaMOX	blaOXA
12	Blood	*A. caviae*			blaMOX	blaOXA
13	Blood	*A. caviae*			blaMOX	blaOXA
14	Wound	*A. caviae*			blaMOX	blaOXA
15	Body Fluid	*A. caviae*			blaMOX	blaOXA
16	Blood	*A. caviae*			blaMOX	blaOXA
17	Stool	*A. caviae*			blaMOX	blaOXA
18	Urine	*A. caviae*			blaMOX	blaOXA
19	Respiratory	*A. media*				blaOXA
20	Wound	*A. dhakensis/A. hydrophila*		cphA		blaOXA
21	Blood	*A. dhakensis/A. hydrophila*		cphA		blaOXA
22	Respiratory	*A. hydrophila*	SHV, AAK, TEM	cphA	CepS	blaOXA (three types)
23	Body Fluid	*A. hydrophila*	SHV, AAK	cphA	CepS	blaOXA (two types)
24	*Body Fluid*	*A. hydrophila*		cphA, VIM	CepS	blaOXA
25	Body Fluid	*A. hydrophila*	KPC, TEM, SHV, AAK	cphA	CepS	blaOXA (two types)
26	Wound	*A. hydrophila*		cphA	CepS	blaOXA
27	Body Fluid	*A. hydrophila*		cphA		blaOXA
28	Wound	*A. hydrophila*		cphA	CepS	blaOXA
29	Urine	*A. hydrophila*		cphA	CepS	blaOXA
30	Wound	*A. hydrophila*		cphA	CepS	blaOXA
31	Blood	*A. veronii*		cphA		blaOXA
32	Blood	*A. veronii*		cphA		blaOXA
33	Blood	*A. veronii*		cphA		blaOXA
34	Blood	*A. veronii*		cphA		blaOXA
35	Blood	*A. veronii*		cphA		blaOXA
36	Stool	*A. veronii*		cphA		blaOXA
37	Blood	*A. veronii*		cphA		blaOXA
38	Blood	*A. veronii*		cphA		blaOXA
39	Respiratory	*A. veronii*		cphA		blaOXA
40	Stool	*A. veronii*		cphA		blaOXA
41	Blood	*A. veronii*		cphA		blaOXA
42	Blood	*A. veronii*		cphA		blaOXA
43	Blood	*A. veronii*		cphA		blaOXA

The presence of beta-lactamase genes is indicated in Table 2. Blank fields indicate a beta-lactamase in that class was not found. All isolates in this study carried a *blaOXA* family Ambler Class D β-lactamase gene. In contrast, the presence of Class B MBL and Class C β-lactamases was species specific. Every isolate of *A. veronii* and *A. hydrophila* encoded a *cphA* family MBL, which was not detected in any isolate of *A. caviae*. Conversely, every isolate of *A. caviae* carried a *blaMOX* family Class C β-lactamase gene, which was not found in other species. Eight out of nine isolates of *A. hydrophila* carried a CepS Class C β-lactamase gene, and three out of nine carried a Class A β-lactamase gene. Both *A. dhakensis* isolates carried a *blaOXA* family and a *cphA* family gene but no Class C β-lactamase gene, similar to the *A. veronii* isolates in this study. The *A. media* isolate carried neither a Class B nor a Class C β-lactamase. These data are consistent with previously published results and are summarized in Table 2. Additional carbapenemase genes, KPC and VIM, were found in one isolate each (Table 2 and Table S1).

All isolates were tested using both BMD and KB for imipenem, ertapenem, and meropenem to evaluate each drug’s ability to predict the presence of a *cphA* gene (Tables 3 and 4). Current breakpoints from CLSI M45 Ed3:2016 (Table 1) were applied. Since previous studies have shown that an increased bacterial inoculum may lead to improved detection of *cphA*-mediated carbapenem resistance using phenotypic methods (9, 13), we also tested each drug with each method with an increased inoculum (1.5 McF for KB, 3 McF for BMD). No isolates lacking a *cphA* gene tested resistant to any carbapenem with any method, though four isolates each tested intermediate to imipenem or ertapenem (Table 3). In contrast, 21 of the 24 *cphA*-positive isolates tested susceptible to at least one carbapenem by standard inoculum KB or BMD. Most of these isolates had phenotypic results that were discordant among the different methods and drugs (Table 4). An increased inoculum improved detection of *cphA-*mediated carbapenem resistance for all drugs using both KB and BMD (Tables 4 and 5). Ertapenem had the highest detection rate at standard inoculums, testing resistant for 5/24 (KB) and 5/24 (BMD) isolates, respectively. Imipenem performed better at higher inoculums, with 14/24 (KB) and 18/24 (BMD) isolates carrying *cphA* testing resistant. Meropenem performed worst for the detection of *cphA-*mediated carbapenem resistance, testing resistant for 3/24 (KB) and 4/24 (BMD) isolates using standard inoculums (Table 5). Average disk zone sizes were smaller and MICs higher for *cphA*-positive isolates (Fig. S2 through S5), but no clear bimodal distribution between *cphA*-positive and *cphA*-negative isolates was observed.

**TABLE 3:** Genotypic and phenotypic susceptibility test results for cphA-negative isolates^*a*^

ID		*cphA* detected	mCIM	eCIM	Imipenem	Ertapenem	Meropenem	Imipenem	Ertapenem	Meropenem	Imipenem	Ertapenem	Meropenem	Imipenem	Ertapenem	Meropenem
					0.5 McF			1.5 McF			0.5 McF			3 McF		
	Species				Kirby-Bauer						Broth microdilution					
01	*A. caviae*	–	–	N/A	S	S	S	S	S	S	S	S	S	S	S	S
02	*A. caviae*	–	–	N/A	S	S	S	S	S	S	S	S	S	S	S	S
03	*A. caviae*	–	–	N/A	S	S	S	S	S	S	S	S	S	S	S	S
04	*A. caviae*	–	–	N/A	S	S	S	S	S	S	S	S	S	S	S	S
05	*A. caviae*	–	–	N/A	S	S	S	S	S	S	S	S	S	S	S	S
06	*A. caviae*	–	–	N/A	S	S	S	S	S	S	S	S	S	S	I	S
07	*A. caviae*	–	–	N/A	S	S	S	S	S	S	S	S	S	S	S	S
08	*A. caviae*	–	–	N/A	S	S	S	S	S	S	S	S	S	S	S	S
09	*A. caviae*	–	–	N/A	S	S	S	S	S	S	S	S	S	S	S	S
10	*A. caviae*	–	–	N/A	S	S	S	I	S	S	S	S	S	S	S	S
11	*A. caviae*	–	–	N/A	S	S	S	S	S	S	S	S	S	S	S	S
12	*A. caviae*	–	–	N/A	S	S	S	S	S	S	S	S	S	S	S	S
13	*A. caviae*	–	–	N/A	S	S	S	S	S	S	S	S	S	S	S	S
14	*A. caviae*	–	–	N/A	S	S	S	S	S	S	S	S	S	S	S	S
15	*A. caviae*	–	–	N/A	S	S	S	S	S	S	S	S	S	S	S	S
16	*A. caviae*	–	–	N/A	S	S	S	S	S	S	S	S	S	S	S	S
17	*A. caviae*	–	–	N/A	I	S	S	S	S	S	S	S	S	S	S	S
18	*A. caviae*	–	–	N/A	I	S	S	S	S	S	S	S	S	S	S	S
19	*A. media*	–	–	N/A	S	S	S	S	S	S	S	S	S	S	S	S

^*a*^ S, susceptible; I, Intermediate; R, Resistant; + indicates positive result; – indicates negative result; N/A indicates not applicable.

**TABLE 4:** Genotypic and phenotypic susceptibility test results for cphA-positive isolates^*a*^

ID	Species	*cphA* detected	mCIM	eCIM	Imipenem	Ertapenem	Meropenem	Imipenem	Ertapenem	Meropenem	Imipenem	Ertapenem	Meropenem	Imipenem	Ertapenem	Meropenem
					0.5 McF			1.5 McF			0.5 McF			3 McF		
					Kirby-Bauer						Broth microdilution					
20	*A. dhakensis/A. hydrophila*	+	+	+	I	S	S	I	S	S	S	S	S	R	R	I
21	*A. dhakensis/A. hydrophila*	+	+	+	I	S	S	I	S	S	S	S	S	R	R	S
22	*A. hydrophila*	+	+	–	I	R	S	R	R	I	I	S	R	R	R	R
23	*A. hydrophila*	+	+	–	R	R	R	R	R	R	R	R	R	R	R	R
24	*A. hydrophila*	+	+	+	R	R	R	R	R	R	R	R	R	R	R	R
25	*A. hydrophila*	+	+	–	R	R	R	R	R	R	R	R	R	R	R	R
26	*A. hydrophila*	+	+	+	I	S	S	R	I	S	S	S	S	I	S	S
27	*A. hydrophila*	+	+	–	S	R	S	I	R	I	I	R	I	R	R	R
28	*A. hydrophila*	+	+	+	I	S	S	I	S	S	S	S	S	R	S	S
29	*A. hydrophila*	+	+	+	I	I	S	R	R	I	I	S	S	I	I	S
30	*A. hydrophila*	+	+	+	I	I	S	I	I	S	S	S	S	R	R	I
31	*A. veronii*	+	+	+	I	S	S	I	S	S	S	S	S	R	R	R
32	*A. veronii*	+	+	+	S	S	S	S	S	S	S	S	S	S	S	S
33	*A. veronii*	+	+	+	I	S	S	R	I	I	I	I	S	R	R	R
34	*A. veronii*	+	+	+	I	S	I	R	I	I	S	R	S	R	R	R
35	*A. veronii*	+	+	+	I	S	S	I	I	S	I	I	S	R	R	R
36	*A. veronii*	+	+	+	I	S	S	R	S	S	S	S	S	R	R	S
37	*A. veronii*	+	+	+	I	S	S	R	I	S	I	S	S	I	I	S
38	*A. veronii*	+	+	+	I	S	S	R	S	S	I	I	I	R	S	R
39	*A. veronii*	+	+	–	I	S	S	R	R	I	S	S	S	R	R	R
40	*A. veronii*	+	+	+	R	I	I	R	R	I	R	S	S	R	S	S
41	*A. veronii*	+	+	+	S	S	S	I	S	S	S	S	S	R	S	S
42	*A. veronii*	+	+	+	I	S	S	R	I	S	S	S	S	S	S	S
43	*A. veronii*	+	+	+	S	S	S	I	S	S	S	S	S	S	S	S

^*a*^ S, susceptible; I, Intermediate; R, Resistant; Gray highlighted boxes indicate discordant phenotypic and genotypic results; + indicates positive result; – indicates negative result; N/A indicates not applicable.

**TABLE 5:** Detection of cphA-encoding isolates using routine and increased-inoculum phenotypic susceptibility testing

*cphA* detection rate		Drug % resistant (number of isolates)		
Method	Inoculum	Imipenem	Ertapenem	Meropenem
Kirby-Bauer	0.5 McF	17% (4/24)	21% (5/24)	13% (3/24)
	1.5 McF	58% (14/24)	33% (8/24)	13% (3/24)
Broth Microdilution	0.5 McF	17% (4/24)	21% (5/24)	17% (4/24)
	3 McF	75% (18/24)	58% (14/24)	46% (11/24)

The mCIM was performed on each isolate, followed by eCIM if the isolate was positive by mCIM (Tables 3 and 4). The mCIM predicted the presence of a *cphA* gene with 100% accuracy (24/24), with each *cphA*-encoding isolate testing positive by mCIM and all *cphA*-negative isolates testing negative (Table 6). In contrast, only 19/24 (79.2%) of *cphA*-positive isolates tested positive by eCIM (Table 4). Since an eCIM can only be interpreted if the mCIM is positive, no mCIM- or *cphA*-negative isolates were tested by eCIM.

**TABLE 6:** Predicting the presence of a cphA gene using the mCIM

		***cphA*** gene		
		Detected	Not detected	
mCIM	Positive	24	0	PPV = 100%
	Negative	0	19	NPV = 100%
		Sensitivity 100%	Specificity 100%	

## DISCUSSION

Although carbapenems are not usually used as first-line treatment for infections with *Aeromonas* spp., they are frequently used as empiric treatment for bacteremia and to treat serious or polymicrobial infections that include *Aeromonas* spp (30, 31). Although a definitive link between *cphA*-type MBL and treatment failure with carbapenems has not been proven, emerging carbapenem resistance and poor outcomes following treatment of *Aeromonas* sp. infections with carbapenems are commonly reported (16, 30–32). Carbapenem resistance mediated by *cphA* has also been shown to be inducible by carbapenem exposure, both through increased expression and mutational changes (33–35). Taken together, these data imply that carbapenem resistance may emerge rapidly in *Aeromonas* spp. carrying *cphA*, increasing the risk for treatment failure and poor outcomes. Failure to detect carbapenem resistance mediated by a *cphA*-type MBL by routine phenotypic methods can give a provider false confidence in the effectiveness of carbapenem treatment for *Aeromonas* spp. infections (16).

In this study, over half of prospectively collected isolates encoded a *cphA* gene, suggesting that there is a high likelihood of encountering a carbapenem-resistant isolate. Routine methods failed to reliably detect carbapenem resistance mediated by *cphA* in all isolates, with only 5/24 isolates testing resistant to ertapenem using KB and BMD. Only three isolates of *A. hydrophila* were resistant to all three carbapenems tested. Most concerning is the inability of broth microdilution to detect *cphA*-mediated carbapenem resistance. This method has long been considered the gold standard of antibiotic susceptibility testing. Previous studies have shown that automated platforms, such as the VITEK2, also fail in detecting *cphA*-mediated carbapenem resistance and have also suggested the use of an alternative phenotypic method (22). Since average MIC values were higher and zone sizes smaller for *cphA*-positive isolates, breakpoints could be adjusted to potentially increase the performance of phenotypic tests. Yet, the absence of a clear bimodal distribution means that no alternative breakpoints could be set to reliably separate *cphA*-positive from *cphA*-negative isolates. Together, these data show that routine susceptibility testing methods are inadequate for reliably detecting *cphA*-mediated carbapenem resistance in patient isolates. This raises serious concerns for the clinical laboratory and the ability to report reliable carbapenem susceptibility results.

Multiple reports have shown that *cphA* is associated with certain species of *Aeromonas* (reviewed in reference 10), raising the possibility of predicting the presence of the gene based on species-level identification. One study found that 100% of *A. hydrophila* (21/21)*, A. veronii* (23/23)*,* and *A. jandaei* (3/3) carried *cphA,* whereas only 1.6% of *A. caviae* (1/62) and no *A. trota* (0/4) or *A. schubertii* (0/1) carried *cphA* (9). Another study showed that 100% of *A. veronii* (21/21), 90% of *A. hydrophila* (18/20), 87.2% of *A. dhakensis* (34/39), 75% of *A. jandaei* (3/4), and 100% of *A. bestiarum* (1/1) carried *cphA*, but 0% of *A. caviae* (0/14) did (11). These findings mirror our own, with all *A. hydrophila, A. veronii,* and *A. dhakensis/A. hydrophila* carrying *cphA*, whereas the gene was not detected in any *A. caviae* isolates. However, these studies also show that the species association is not perfect. Furthermore, species-level identification of *Aeromonas* spp. is not trivial, as MALDI-ToF and *rrs* sequence analysis fail to provide reliable identification (11, 15, 23). Despite these data, identification methods, such as MALDI-ToF, may be accurate enough to serve as a preliminary screening tool for *cphA*-positive *Aeromonas* sp. Laboratories may consider evaluating their identification method for the ability to rule in or rule out *cphA*-positive species.

Studies have consistently shown that carbapenemase screening methods such as the CarbaNP test perform well for the detection of *cphA* (11, 36). It is not clear why these methods perform better than routine phenotypic testing, but it is possible that these methods better allow *Aeromonas* spp. to initiate expression of *cphA* before the organism is completely eliminated. The mCIM is another reliable method for detecting carbapenemase production in carbapenem-resistant *Enterobacterales* (37). Previous work has shown promising results using mCIM for detection of carbapenemases, including non-*cphA* carbapenemases, in *Aeromonas* sp (22). Our study shows that the mCIM is also able to detect carbapenem resistance due to *cphA* in *Aeromonas* spp. with high sensitivity and specificity, suggesting that although it is not currently endorsed for use in *Aeromonas* spp. by CLSI, this test may play a role in susceptibility testing for these organisms.

In contrast, the eCIM, which is used for the differentiation of MBL from serine carbapenemases, failed to accurately identify five *cphA*-positive isolates in our study, similar to another study where only 29/33 *cphA*-carrying, mCIM-positive isolates were positive by eCIM (21). Two isolates had an additional carbapenemase, VIM and KPC. Both were mCIM positive. As expected, the isolate carrying a KPC gene was negative by eCIM. Although there are reports of carbapenemases other than *cphA* in *Aeromonas* spp. isolates (38, 39), these remain rare in clinical isolates (40), so differentiation of MBL by eCIM may not be important for clinical management at this point.

Using a higher inoculum for broth microdilution and disk diffusion was not a viable alternate strategy in this study; even though separation between zone sizes and MICs of cphA-positive and cphA-negative populations was better with an increased inoculum, the overall sensitivity and specificity was still poor. This method is not endorsed for any susceptibility testing by CLSI, and CLSI breakpoints should not be used clinically outside of the specific conditions for which they were developed, including the inoculum size. Although standardized methods and breakpoints could be developed using an increased inoculum, the lack of a clear bimodal MIC or zone size distribution at higher inocula suggests that there may be limited value in pursuing this approach. Another method that may improve detection of *cphA-*mediated carbapenem resistance by phenotypic tests is the induction of *cphA* expression prior to testing, such as using colonies from a beta-lactam disk zone edge. This strategy was not evaluated in this study, but induction of imipenem degradation mediated by cphA after exposure to benzylpenicillin has been shown (12)

Carbapenem resistance mediated by *cphA* is common in *Aeromonas* spp. and is difficult to detect using routine methods. Laboratories should consider not reporting carbapenems as susceptible for these organisms unless the presence of *cphA* has been excluded. Since carbapenems are not first-line treatment for *Aeromonas* spp., this additional testing could be performed only on specific specimen sources, or upon request. Whereas *cphA* could be detected using molecular methods such as WGS, no commercial assays are currently available, and developing and routinely using molecular methods for *cphA* detection is cost-prohibitive for broad adoption. The mCIM is a simple and reliable method for the detection of carbapenemases, including *cphA*. Laboratories may consider validating the mCIM for the routine detection of *cphA*-mediated carbapenem resistance in clinical isolates of *Aeromonas* spp. prior to the release of carbapenem susceptibility results. There is currently limited data on carbapenem resistance mechanisms in *Aeromonas* spp. other than carbapenemases, but any carbapenemase test should be supplemented by routine phenotypic testing to rule out carbapenem resistance by other mechanisms in carbapenemase-negative isolates. If the laboratory can reliably distinguish between the species of *Aeromonas* with and without a *cphA* gene, this identification could potentially also be used to guide further testing and reporting.

## SUPPLEMENTAL MATERIAL

Supplemental figures jcm.01272-25-s0001.pdf Figures S1 to S5.

Table S1 jcm.01272-25-s0002.xlsx Genotypic and phenotypic data for each *Aeromonas* isolate.

ASM does not own the copyrights to Supplemental Material that may be linked to, or accessed through, an article. The authors have granted ASM a non-exclusive, world-wide license to publish the Supplemental Material files. Please contact the corresponding author directly for reuse.
