# Supplementary material for: Discrepancy between reference phenotypic and genotypic detection of cphA metallo-β-lactamase in Aeromonas spp
Source: J Clin Microbiol. 2026 Apr 30;64(6):e01272-25. doi: 10.1128/jcm.01272-25 (PMC13251357; doi:10.1128/jcm.01272-25)
Supplement: Supplemental figures — Figures S1 to S5. [file jcm.01272-25-s0001.pdf]

Supplemental Figure 1. Kmer Tree for species identification

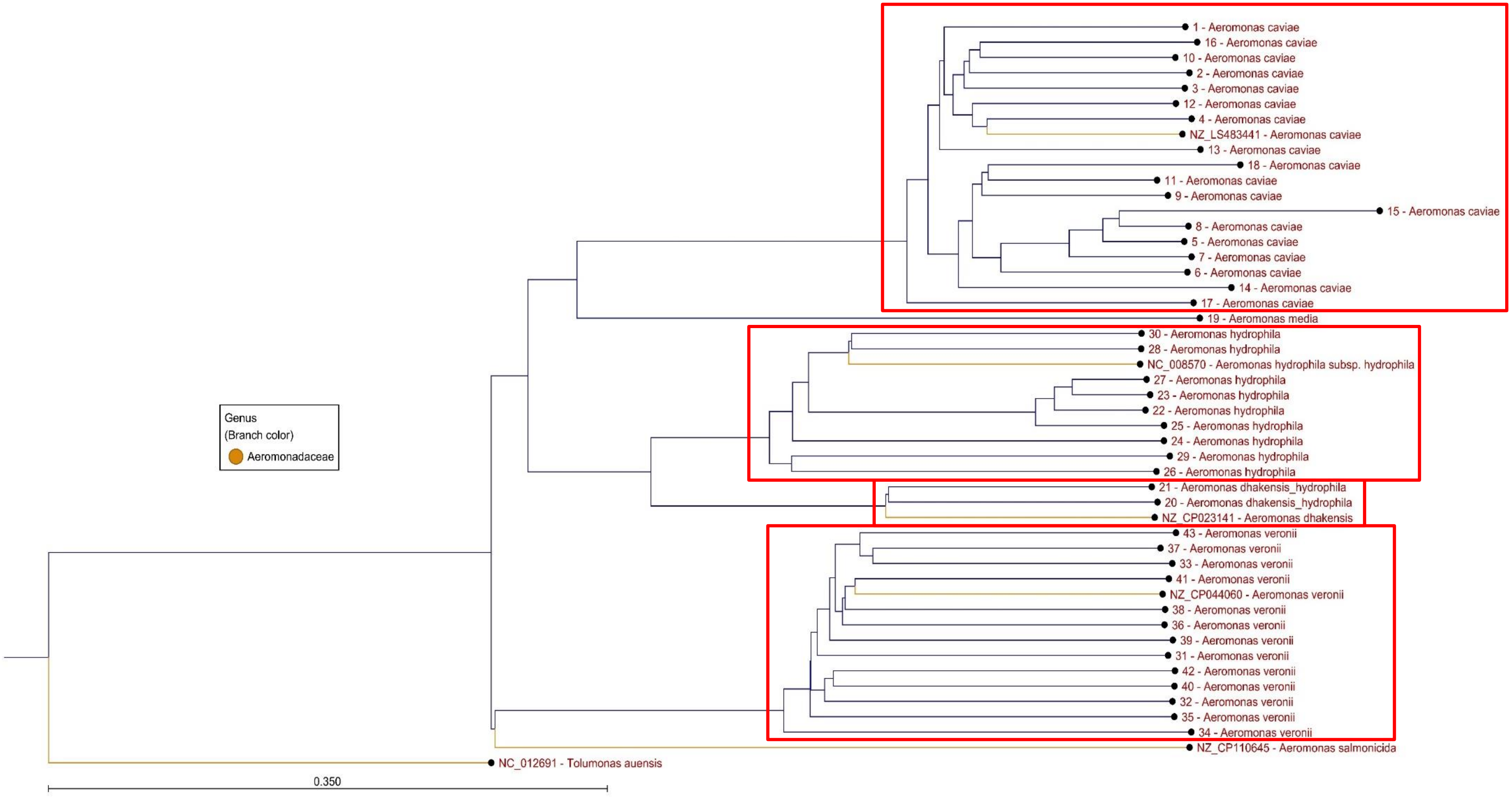

Supplemental Figure 1: Kmer analysis was performed with the 43 study isolate (labeled 1 through 43 with species name) and 6 reference genomes. These reference genomes are NC_012691 Tolumonas auensis (used as an outgroup), Aeromonas salmonicida NZ_CP110645, Aeromonas veronii NZ_CP044060, Aeromonas dhakensis NZ_CP023141, Aeromonas hydrophila NC_008570, and Aeromonoas caviae NZ_LS483441.

Supplemental Figure 2.

A.

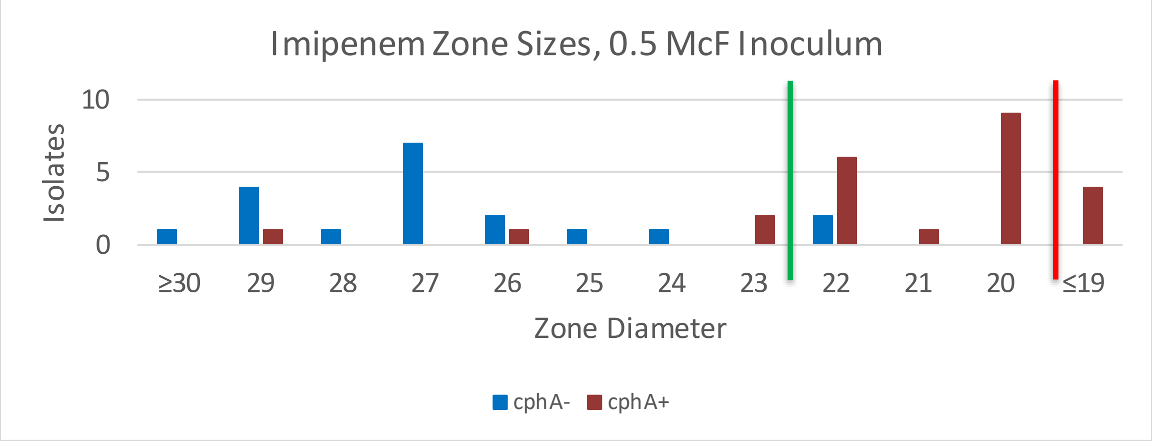

B.

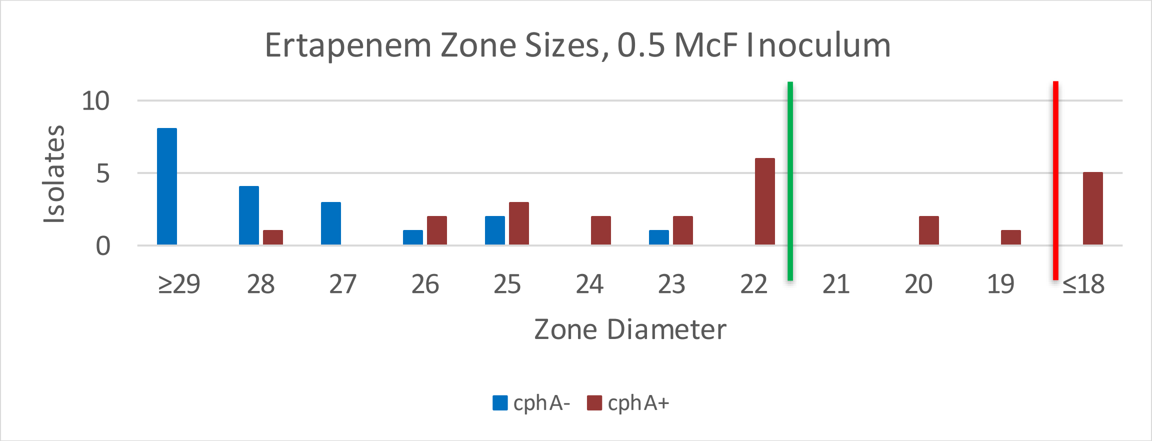

C.

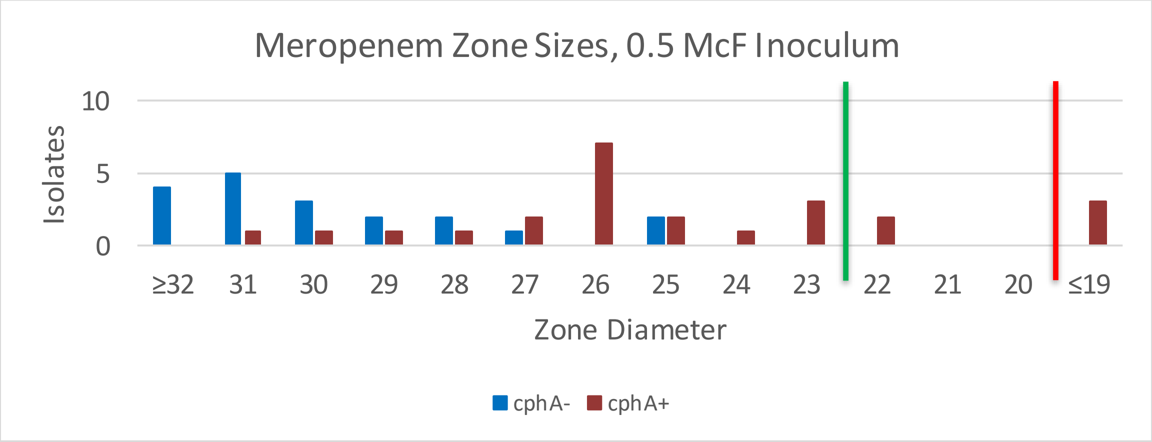

Supplemental Figure 2: Disk zone sizes using a 0.5 McF inoculum. Average zone size for cphA-negative compared to cphA-positive isolates were 26.9 mm, and 28.0 mm vs. 30.0 mm vs. 19.6 mm vs. 19.9 mm, 22.9 mm for (A) imipenem, (B) ertapenem, and (C) meropenem, respectively. Resistant (red) and susceptible (green) breakpoints are delineated with vertical lines.

Supplemental Figure 3.

A.

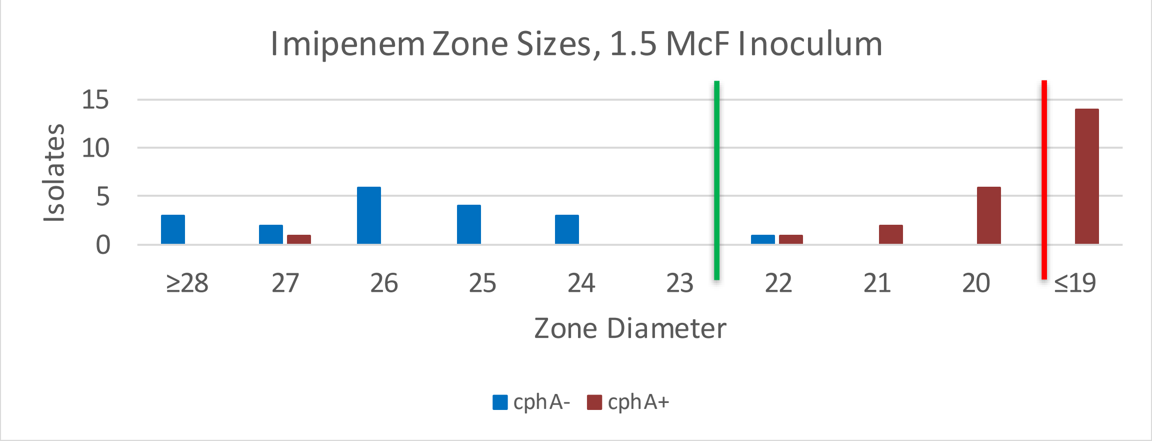

B.

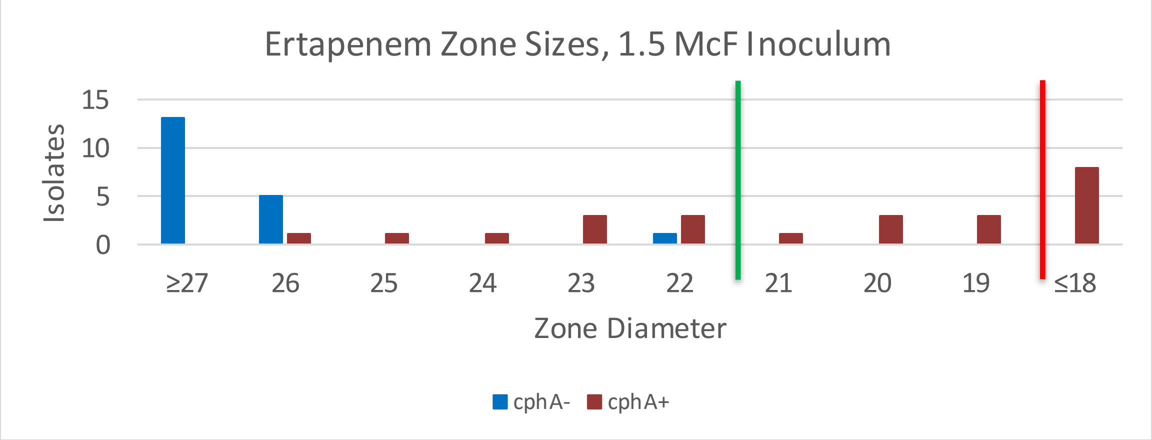

C.

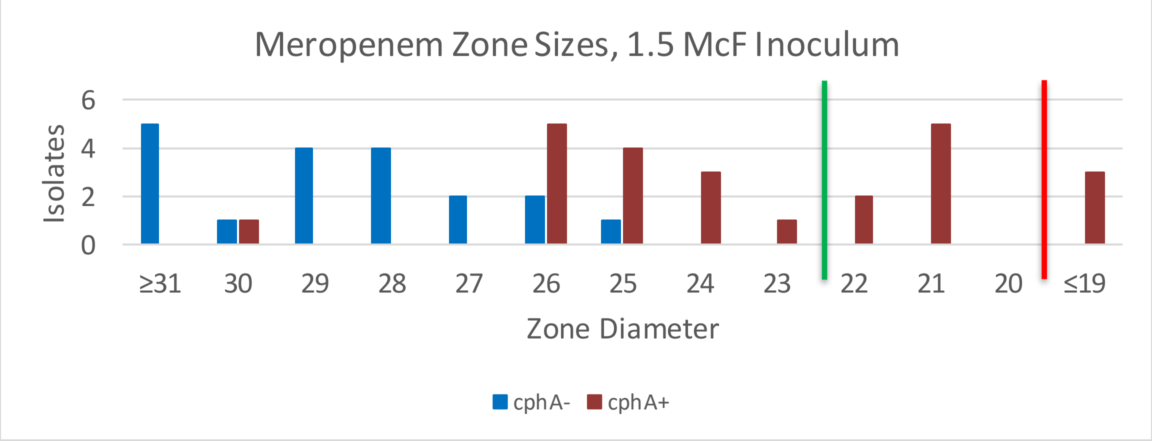

Supplemental Figure 3: Disk zone sizes using a 1.5 McF inoculum. Average zone size for cphA-negative compared to cphA-positive isolates were 25.8 mm vs. 27.2 mm, 28.8 mm vs. 17.9 mm, and 18.0 mm vs. 21.4 mm for (A) imipenem, (B) ertapenem, and(C) meropenem, respectively. Resistant (red) and susceptible (green) breakpoints are delineated with vertical lines.

Supplemental Figure 4.

A.

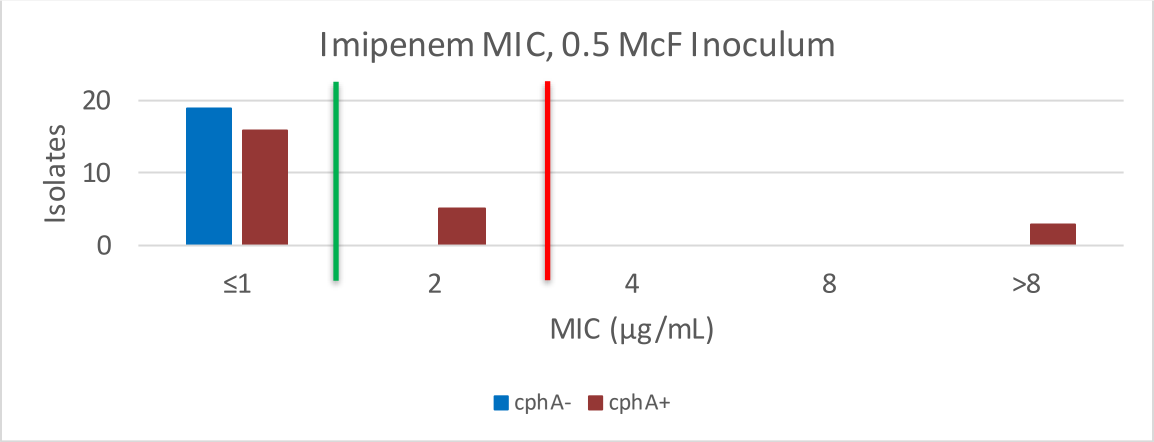

B.

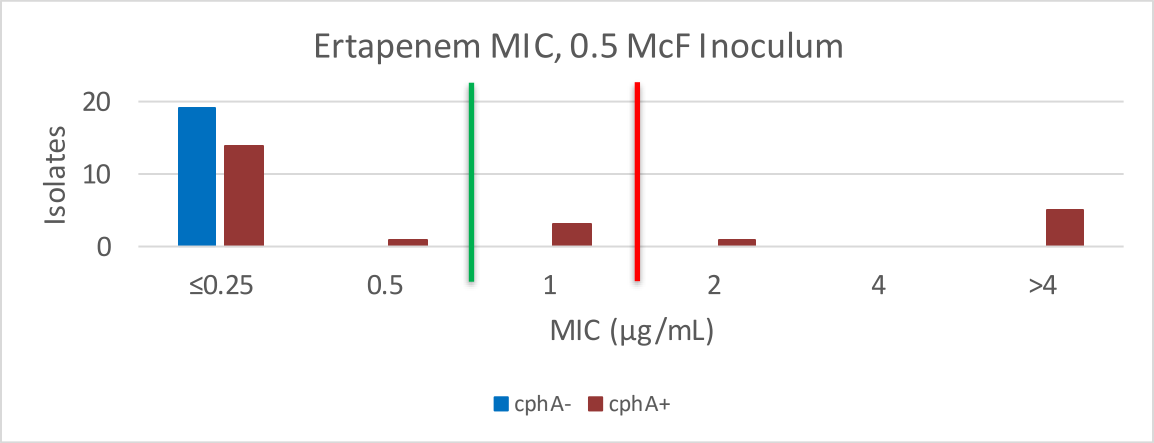

C.

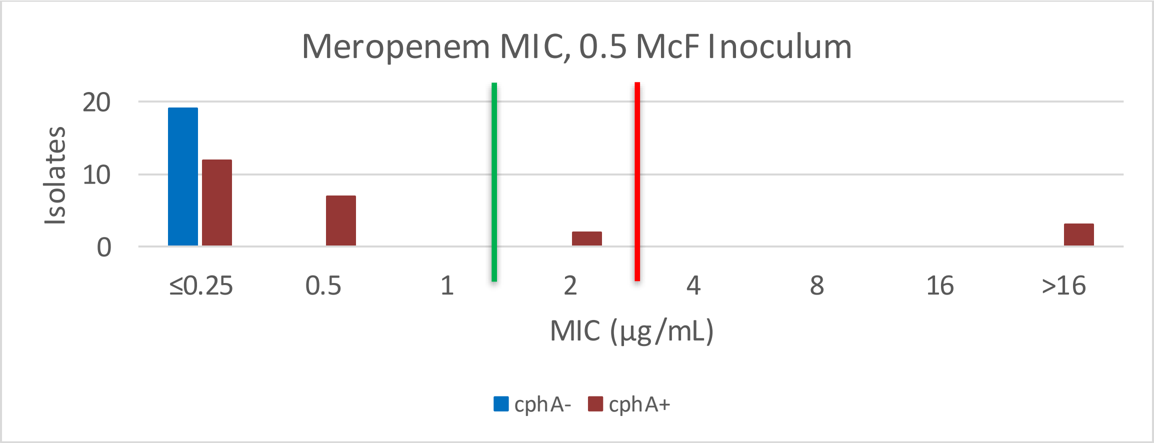

Supplemental Figure 4: MICs for (A) imipenem, (B)ertapenem, and (C) meropenem using a 0.5 McFarland inoculum. Resistant (red) and susceptible (green) breakpoints are delineated with vertical lines.

Supplemental Figure 5.

A.

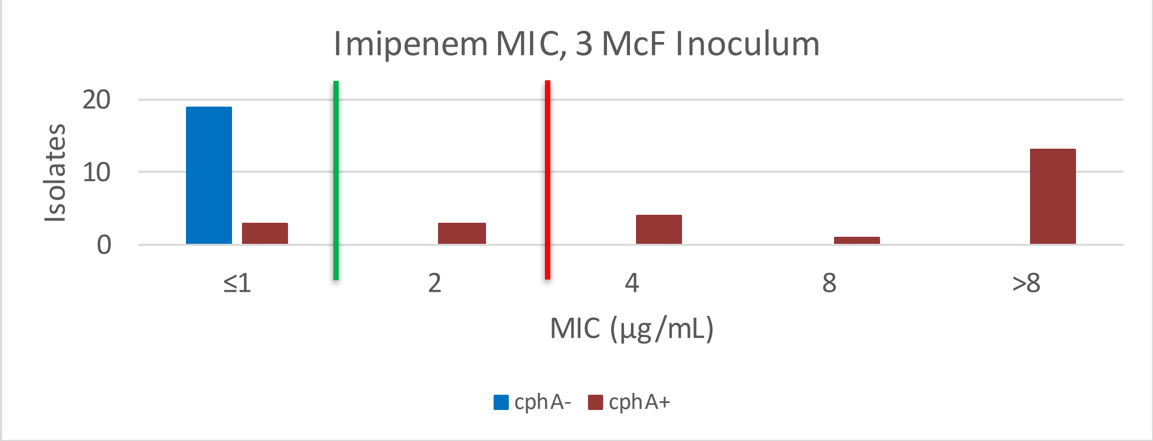

B.

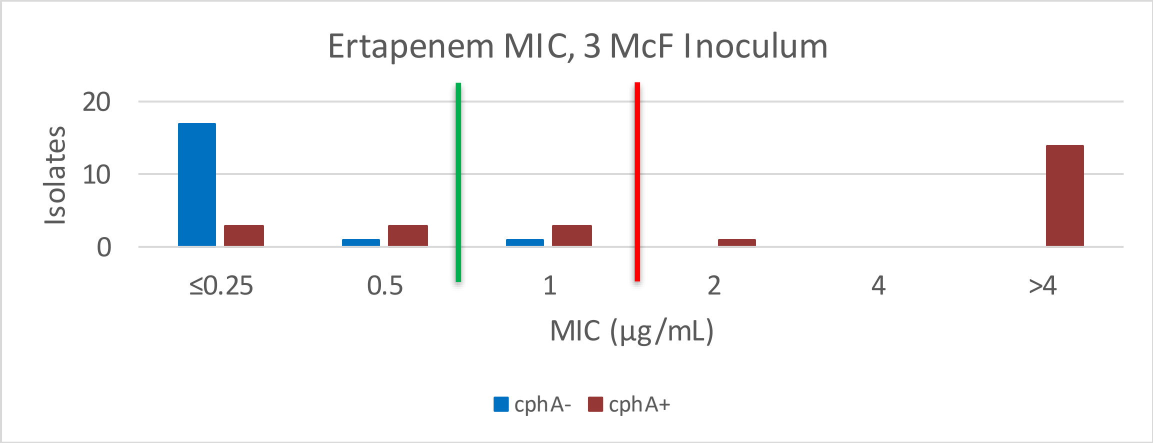

C.

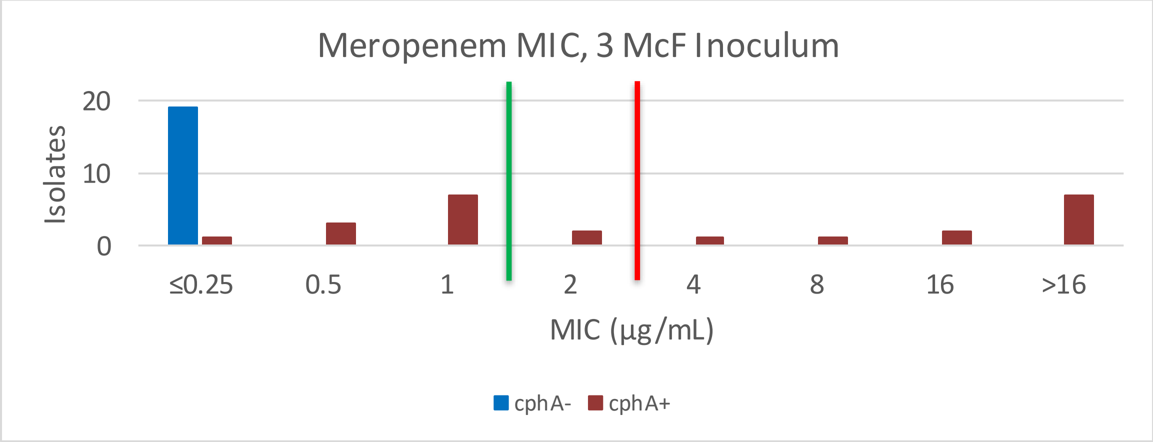

Supplemental Figure 5: MICs for (A) imipenem, (B) ertapenem, and (C) meropenem using a 3 McFarland inoculum. Resistant (red) and susceptible (green) breakpoints are delineated with vertical lines.
